# Efficacy and safety of anticoagulation in atrial fibrillation patients with intracranial hemorrhage: A systematic review and meta-analysis

**DOI:** 10.3389/fphar.2023.1122564

**Published:** 2023-03-09

**Authors:** Qiang Zhou, Xiang Liu, Xian Yang, Xiao-Hui Huang, Yan-Zi Wu, Ying-Ying Tao, Meng Wei

**Affiliations:** ^1^ Department of Clinical Pharmacy, Jinling Hospital, Medical School of Nanjing University, Nanjing, China; ^2^ Department of Pharmacy and Traditional Chinese Pharmacy, Jiangsu College of Nursing, Huaian, China; ^3^ Department of Pharmacy, Nanjing Drum Tower Hospital, The Affiliated Hospital of Nanjing University Medical School, Nanjing, China

**Keywords:** atrial fibrillation, intracranial hemorrhage, oral anticoagulant therapy, meta-analysis, non-vitamin K antagonist oral anticoagulants

## Abstract

**Background:** The benefits and risks of starting anticoagulation therapy, such as direct oral anticoagulations (DOACs) or warfarin, in atrial fibrillation (AF) patients with a history of intracranial hemorrhage (ICH) remain controversial. We performed a systematic review and meta-analysis to compare the safety and efficacy of starting oral anticoagulation (OAC) and non-oral anticoagulation in these patients.

**Methods:** PubMed, Cochrane Library, and Embase were searched from inception to 01 May 2022 for randomized controlled trials and cohort studies, reporting effectiveness and safety outcomes for anticoagulation therapy in atrial fibrillation patients with intracranial hemorrhage. The Newcastle-Ottawa Scale (NOS) and the Cochrane Collaboration tool were used to evaluate bias risks for all randomized controlled trials (RCTs) and cohort studies. An effects model was applied to calculate adjusted hazard ratios (aHRs) for randomized controlled trials and cohort studies.

**Results:** We analyzed data from two randomized controlled trials (304 patients) and seven Cohort studies (17,477 patients). Compared to non-oral anticoagulation, starting oral anticoagulation therapy reduced the risk of Ischemic Stroke/Systemic Embolism (SE) (aHR: 0.64, 95% CI: 0.55–0.57) and all-cause death (aHR: 0.53, 95% CI: 0.35–0.80) in atrial fibrillation patients and a prior history intracranial hemorrhage. Starting oral anticoagulation therapy did not increase the risk of recurrent intracranial hemorrhage (aHR: 1.07, 95% CI: 0.66–1.74), but increased the risk of major bleeding (aHR: 1.38, 95% CI: 1.00–1.91) than no oral anticoagulation therapy. The DOACs had a lower risk of Ischemic Stroke/SE (aHR: 0.84, 95% CI: 0.70–1.00), recurrent intracranial hemorrhage (aHR: 0.63, 95% CI: 0.49–0.82), and all-cause death (aHR: 0.65, 95% CI: 0.48–0.88) compared to warfarin. According to subgroup analyses, starting oral anticoagulation therapy have a higher risk of recurrent intracranial hemorrhage than non-oral anticoagulation therapy (aHR: 1.57, 95% CI: 1.36–1.81) for Asians.

**Conclusion:** After intracranial hemorrhage in atrial fibrillation patients, restarting or initiating oral anticoagulation therapy decreased the risk of Ischemic Stroke/SE and all-cause death but did not increase the risk for recurrent intracranial hemorrhage. Direct oral anticoagulations have better efficacy and safety than warfarin if oral anticoagulation therapy is started. However, starting oral anticoagulation increases the risk for recurrent intracranial hemorrhage in the Asian region.

## Introduction

Atrial fibrillation (AF) is the most prevalent arrhythmia encountered by clinicians, with a prevalence of approximately 1% worldwide. The prevalence increases significantly with age, reaching up to 9% and 22% in people aged 75 years and 80 years, respectively (Peters and Woodward, 2019). Oral anticoagulation (OAC) is commonly used to prevent stroke events and reduce all-cause death. Intracranial hemorrhage (ICH) remains the most feared complication of OAC therapy. The death occurs within 30 days of the event in approximately 35%–52% of cases (van Asch et al., 2010). Balancing the risks of thromboembolic and bleeding events is a key consideration in AF and a prior history of ICH. It is a challenging issue in clinical treatment if anticoagulation is needed (Hemphill et al., 2015). Patients with a history of ICH have been universally excluded from these randomized controlled trials, leading to a lack of high-level evidence-based medical research findings (Connolly et al., 2009; Granger et al., 2011; Patel et al., 2011). In retrospective studies, Nielsen et al. discovered that starting OAC therapy after ICH decreases the thromboembolism risk and does not increase the incidence of recurrent ICH (Nielsen et al., 2015). In contrast, another study discovered that starting OAC therapy after ICH decreases the thromboembolism risk but increases the incidence of recurrent ICH (Chao et al., 2016). Therefore, it is inconclusive whether oral anticoagulation is superior to avoidance of anticoagulation. In addition, in terms of anticoagulant drug selection, compared to warfarin, DOACs have become the drug choice for general patients with non-valvular AF, but the effectiveness and safety in patients with AF who have a history of ICH is still unclear (Greenberg et al., 2022). As a result, we conducted a meta-analysis to evaluate the effectiveness and safety of anticoagulant therapy for patients with AF who have a history of ICH.

## Methods

### Data sources and searches

The PRISMA Statement was followed for reporting systematic reviews. The PubMed, Embase, and Cochrane Library were searched from inception till 1 May 2022, with the language restricted to English. The search keywords included: “restarting or initiation anticoagulation,” “starting anticoagulation,” “no anticoagulation,” “avoid anticoagulation,” “non-vitamin K antagonist oral anticoagulants,” “direct oral anticoagulant,” “rivaroxaban,” “dabigatran,” “apixaban,” “edoxaban,” “warfarin,” “vitamin K antagonist,” “atrial fibrillation,” “intracranial hemorrhage,” and “intracerebral hemorrhage.”

### Inclusion and exclusion criteria

Eligible studies include 1) patients (≥18 years) with non-valvular AF in combination with ICH (intracerebral, subarachnoid, subdural, and intracranial hemorrhage), 2) studies with starting OAC (warfarin or/and DOACs) or non-OAC for stroke prevention in AF, 3) randomized controlled trials or cohort studies. Exclusion criteria: 1) duplicate published articles, 2) case report or case series, systematic reviews, and meta-analysis, conference or meeting abstracts, 3) no outcomes available, 4) patients with valvular AF, including severe native valve disease and prosthetic mechanical heart valve, and 5) articles that did not report adjusted hazard ratios (aHR) data.

### Outcome definition

The primary outcomes were Ischemic Stroke/SE and recurrent ICH. Secondary outcomes were all stroke, major bleeding, and all-cause death. The definition of major bleeding was accepted by the International Society on Thrombosis and Haemostasis that fall in hemoglobin level ≥2 g/dL, transfusion of at least 2 units of blood, or symptomatic bleeding in a critical area or organ (Schulman et al., 2005). Separate ischemic stroke or systemic embolism outcomes were used instead if no data on Ischemic Stroke/SE.

### Data extraction

The literature and extracted data were screened by two researchers (Q. Z and X. L) independently by the above criteria. Then, all the selected studies were cross-checked by two reviewers. Any objections would be addressed by the third reviewer (X. Y). The extracted information includes the first author’s name, country or region, year of publication, study type, mean age, gender ratio, sample sizes for each group, CHA2DS2-VASc score, HAS-BLED score, history of previous ischemic stroke/transient ischemic attack (TIA), outcome measures (aHR for randomized controlled trials (RCTs) and cohort studies), and longer follow-up.

### Quality assessment

Cochrane Collaboration’s tool and Newcastle-Ottawa Scale (NOS) were used to evaluate the quality of RCTs and cohort studies, respectively (Higgins and Green, 2008; Wells et al., 2010). Cochrane Collaboration’s tool was used to assess six aspects of study quality (selection bias, performance bias, detection bias, attrition bias, reporting bias, and other bias). Low, unclear, or high risk of bias was assigned to each item. NOS quality assessment included three main aspects: selection, comparability, and outcome. The maximum score for each study was nine; studies with scores below seven were considered to have a higher risk of bias.

### Data analysis

This systematic review was reported using the PRISMA statement (Liberati et al., 2009). Software STATA14.0 was used for data analysis. The adjusted hazard ratios (aHRs) and associated 95% confidence intervals (95% CI) were used as the general measure. The heterogeneity test of included studies was evaluated using the Chi-square test and I^2^ statistic. A fixed-effects model was chosen when there was low heterogeneity between studies (I^2^ ≤ 50%), otherwise, the random-effects model was adopted (I^2^ > 50%). Additionally, subgroup analyses of primary outcomes were performed according to different regions (Asian or non-Asian) and study types (RCTs or cohort studies). Sensitivity analysis was conducted using sequential elimination of each study from the pool to test the robustness of the results. The *p*-value <0.05 was considered statistically significant.

## Results

### Study selection

**FIGURE 1:**
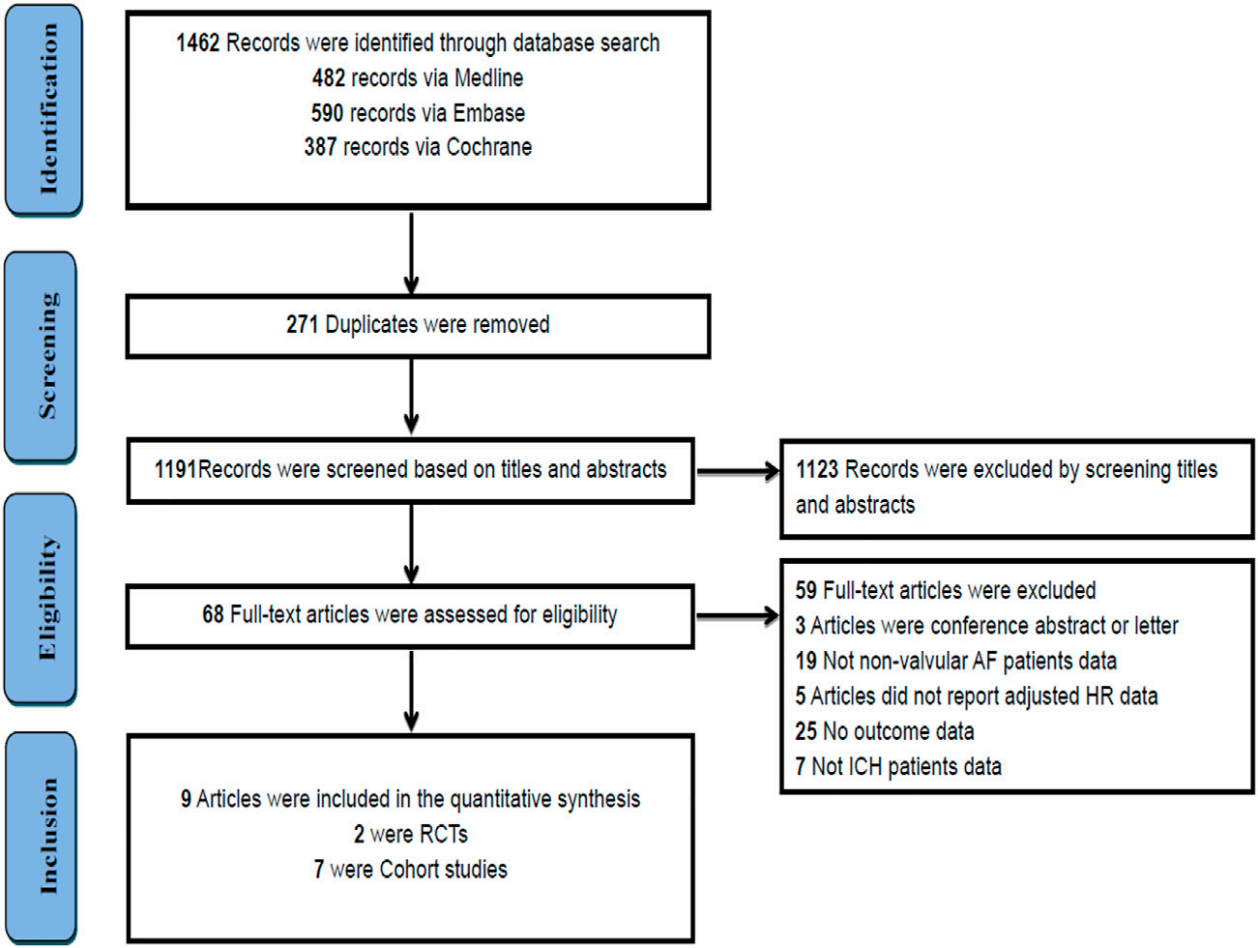
PRISMA screening flow chart.

A total of 1,462 studies were initially identified through a comprehensive search. After eliminating duplicate studies, 271 remained. After screening the titles and abstracts, 68 studies met the requirements. Finally, we included nine studies by reading the full text. Figure 1 presents an overview of the literature selection process.

### Baseline characteristics

Nine publications (Nielsen et al., 2015; Chao et al., 2016; Nielsen et al., 2017; Lee et al., 2020; Newman et al., 2020; Tsai et al., 2020; Schreuder et al., 2021; SoSTART Collaboration, 2021; Lin et al., 2022) included 17,781 patients. Seven studies compared the effectiveness and safety of starting OAC and non-OAC after ICH in AF patients (Nielsen et al., 2015; Chao et al., 2016; Nielsen et al., 2017; Newman et al., 2020; Schreuder et al., 2021; SoSTART Collaboration, 2021; Lin et al., 2022). Three studies compared the effectiveness and safety of warfarin and DOACs (Lee et al., 2020; Tsai et al., 2020; Lin et al., 2022). Lin SY et al. compared the effectiveness and safety of starting OAC and non-OAC, as well as warfarin and DOACs. Our studies included two RCTs (Schreuder et al., 2021; SoSTART Collaboration, 2021), one prospective cohort study (Tsai et al., 2020), and six retrospective cohort studies (Nielsen et al., 2015; Chao et al., 2016; Nielsen et al., 2017; Lee et al., 2020; Newman et al., 2020; Lin et al., 2022). The follow-up period ranged from 0.5 to 3 years Table 1 illustrates the main study characteristics of the publications.

**TABLE 1:** Baseline characteristics of included studies.

The first author	Country or region	Study design	Comparison	Sample size	Age (years)	Male (%)	CHA2DS2-VASc score	HAS-BLED score	History of previous ischemic Stroke/TIA (%)	Follow-up (years)	Outcomes
Year											
OAC vs. Non-OAC											
Lin SY	Taiwan, China	Retrospective cohort	Non-OACs	1,069	76.31	57.81	5.2	N/A	43.97	0.5 and 0.7	1, 2, 4, 5
(2022) Lin et al. (2022)			DOACs or warfarin	283	75.61	58.66	5.31	N/A	51.24		
Schreuder	Holland	RCT	Non-OACs	51	79	55	4	N/A	Ischemic Stroke:27	1.9	1, 2, 4+
(2021) Schreuder et al. (2021)			Apixaban	50	77	54	4	N/A	Ischemic Stroke:20		
SoSTART	United Kingdom	RCT	Non-OACs	102	79	65	4	2	Ischemic Stroke:26, TIA:18	1–2	2, 3
(2021) SoSTART Collaboration (2021)			DOACs or warfarin	101	79	62			Ischemic Stroke:17, TIA:12		
Newman TV	United States of America	Retrospective cohort	Non-OACs	526	N/A	56.27	N/A	N/A	52.09	2.14	1, 2, 5
(2020) Newman et al. (2020)			DOACs	976	N/A	56.25			43.14		
Nielsen PB	Denmark	Retrospective cohort	Non-OACs	2,415	77.1	61.3	3.6	3.9	Ischemic Stroke:29.9	0.8	1, 2, 3, 5
(2017) Nielsen et al. (2017)			warfarin								
Chao TF	Taiwan, China	Retrospective cohort	Non-OACs	1,134	70.5 ± 12.3	54	6	N/A	65	N/A	1, 2
(2016) Chao et al. (2016)			warfarin	1,134	69.5 ± 12.4	54	6	N/A	66		
Nielsen PB	Denmark	Retrospective cohort	Non-OACs	1752	78	62	3.9	3.2	N/A	1	1, 2, 4, 5
(2015) Nielsen et al. (2015)			DOACs or warfarin								
NOAC vs. non-vitamin K											
Lin SY	Taiwan, China	Retrospective cohort	warfarin	205	74.34	57.07	4.92	N/A	40	0.5	1, 2, 4, 5
(2022) (Lin et al., 2022)			DOACs	333	76.23	57.36	5.16	N/A	45.65		
Tsai CT	Taiwan, China	Prospective cohort	warfarin	973	75.5	55.8	5.44	4.29	69.2	N/A	1, 2, 4, 5
(2020) Tsai et al. (2020)			DOACs	973	75.7	51.6	4.59	4.35	69.7		
Lee SR	Korea	Retrospective cohort	warfarin	2,438	72.5 ± 10.2	57.2	4.0 ± 1.6	4.4 ± 1.1	Ischemic Stroke:20.4	0.6	1, 2, 5
(2020) Lee et al. (2020)			DOACs	3,266	72.5 ± 9.9	57.2	4.0 ± 1.6	4.4 ± 1.2	Ischemic Stroke:20.6		

Note: Outcomes, 1, Ischemic Stroke/SE; 2, recurrent ICH; 3, all stroke; 4, major bleeding; 5, all-cause mortality.

### Quality assessment

**FIGURE 2:**
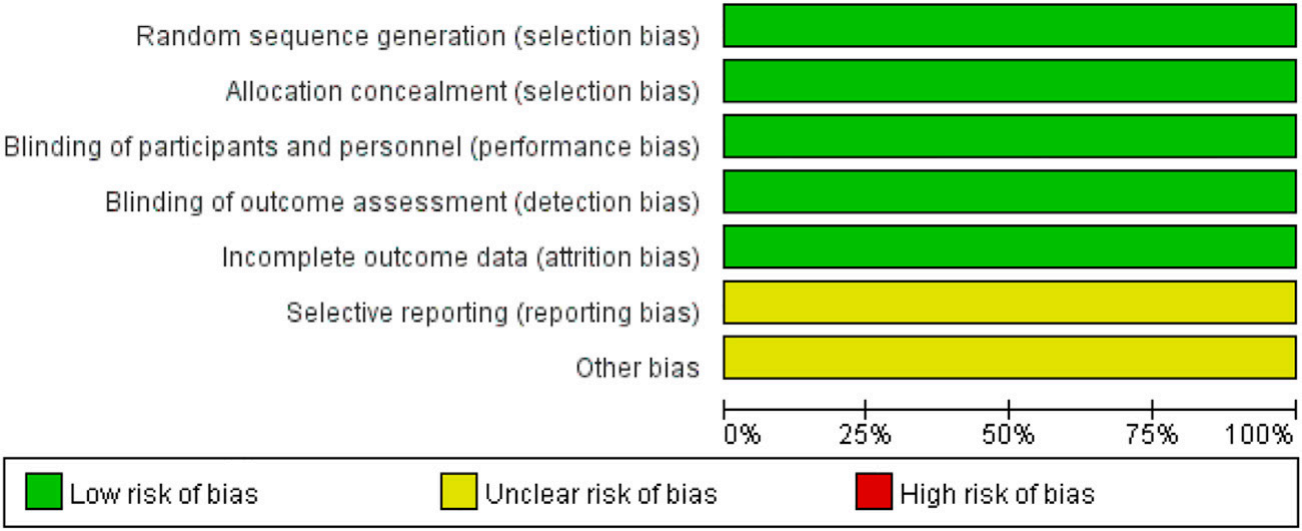
Risk of bias graph.

**FIGURE 3:**
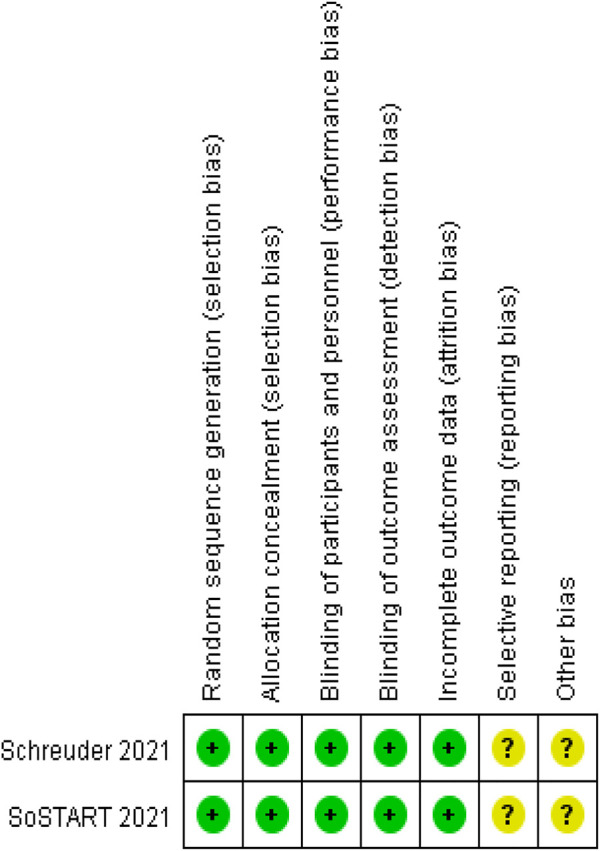
Risk of bias summary.

We analyzed the risk of bias in the methodology of two RCTs using Cochrane 5.1.0 (Schreuder et al., 2021; SoSTART Collaboration, 2021). The randomization method, allocation concealment, and blinding were described for the two included studies. Data profiles were relatively complete for all studies, but selective reporting and other sources of bias were unclear. Seven cohort studies were conducted using the NOS (Nielsen et al., 2015; Chao et al., 2016; Nielsen et al., 2017; Lee et al., 2020; Newman et al., 2020; Tsai et al., 2020; Lin et al., 2022). Each study was classified as high quality (7–9) based on the total score of the test. Figures 2, 3 and Table 2 present the overall risk of bias.

**TABLE 2:** Quality assessment results of cohort studies.

First author, year	Selection	Comparability	Outcome	Score
Lin SY, 2022 Lin et al. (2022)	****	**	***	9
Newman TV, 2020 Newman et al. (2020)	****	*	***	8
Tsai CT, 2020 Tsai et al. (2020)	****	**	*	7
Lee SR, 2020.Lee et al. (2020)	****	**	**	8
Nielsen PB, 2017 Nielsen et al. (2017)	****	*	***	8
Chao TF, 2016 Chao et al. (2016)	****	**	**	8
Nielsen PB, 2015 Nielsen et al. (2015)	****	*	**	7

## Comparing the effectiveness and safety of OAC *versus* non-OAC

### Ischemic stroke/SE

**FIGURE 4:**
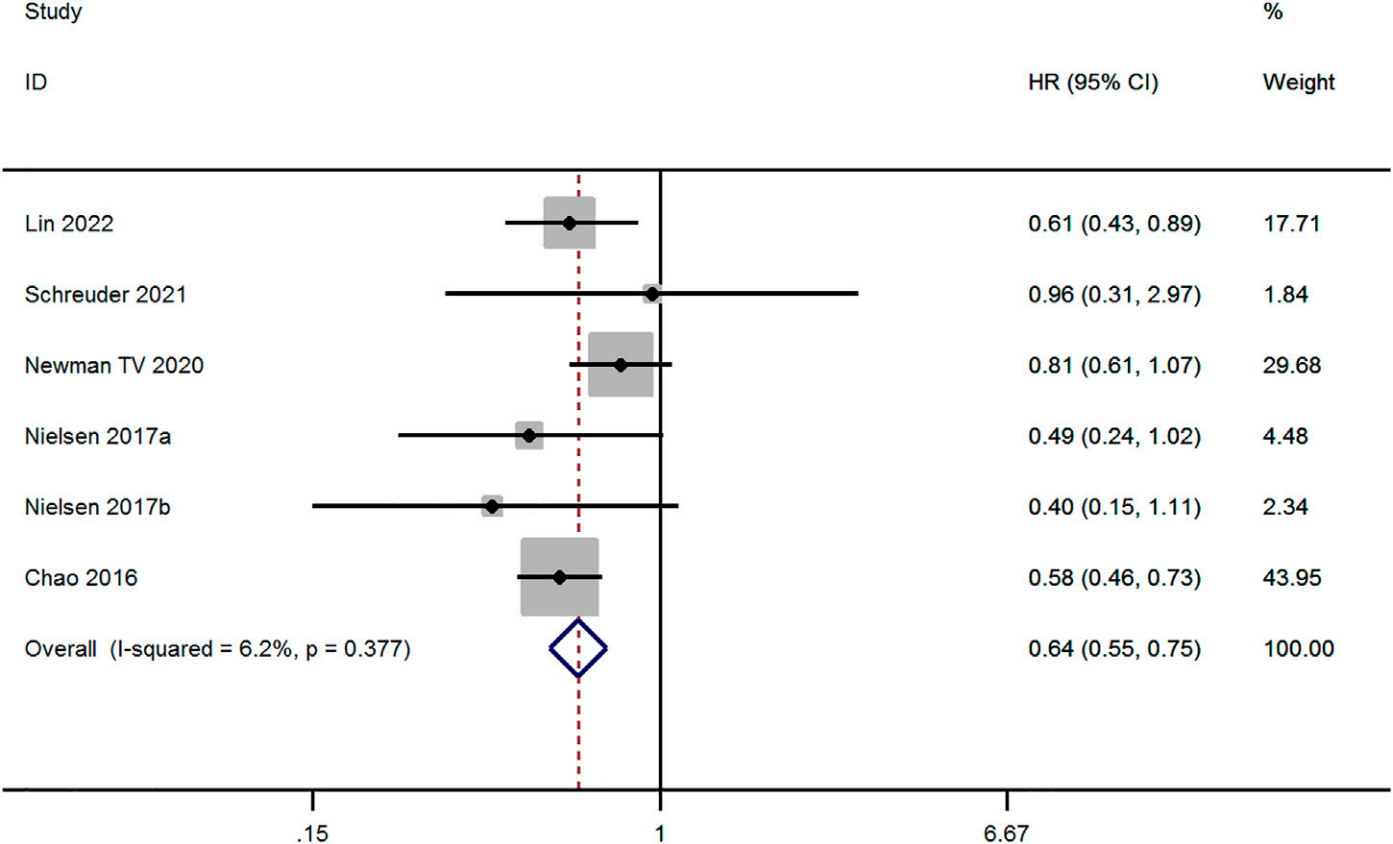
Meta-analysis of Ischemic Stroke/SE risk in OAC *versus* non-OAC.

Six studies evaluated the risk of Ischemic Stroke/SE between OAC and non-OAC in AF patients with ICH (Chao et al., 2016; Nielsen et al., 2017; Newman et al., 2020; Schreuder et al., 2021; Lin et al., 2022). The fixed-effect model was applied to studies with low heterogeneity (*p* ≥ 0.1 and I^2^ ≤ 50%). The result demonstrates that Ischemic Stroke/SE risk for OAC therapy was lower than for non-OAC therapy (aHR, 0.64; 95% CI, 0.55–0.75, *p* < 0.001, Figure 4).

### Recurrent ICH

**FIGURE 5:**
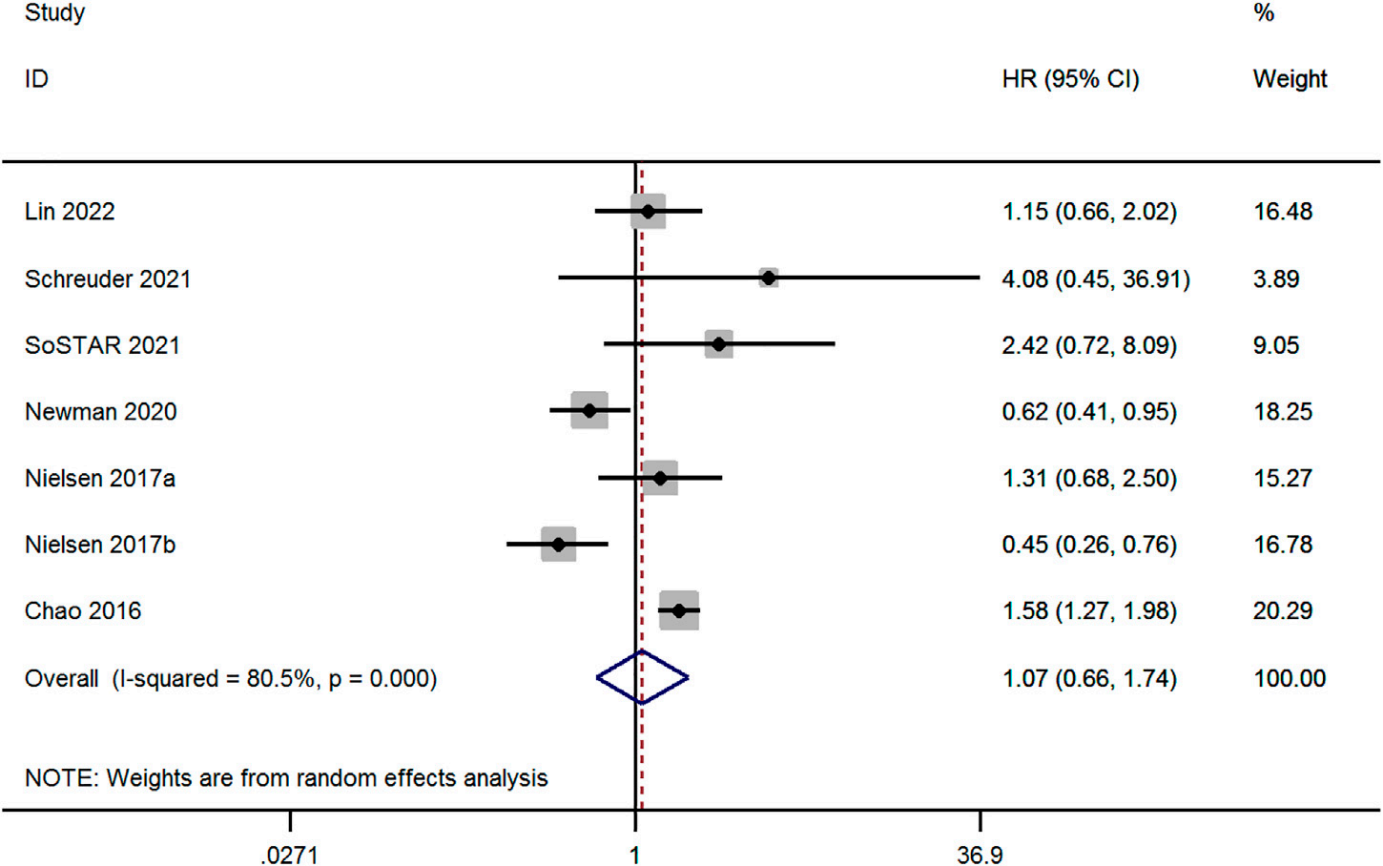
Meta-analysis of the risk of recurrent ICH in OAC *versus* non-OAC.

Seven studies evaluated the risk of recurrent ICH between OAC and non-OAC in AF patients with ICH (Chao et al., 2016; Nielsen et al., 2017; Newman et al., 2020; Schreuder et al., 2021; SoSTART Collaboration, 2021; Lin et al., 2022). High heterogeneity was observed among the studies (*p* < 0.1 and I^2^ > 50%), the random-effect model was adopted, and there was no significant difference among the studies (aHR, 1.07; 95% CI, 0.66–1.74, *p* = 0.774, Figure 5).

### Major bleeding, all stroke, and all-cause mortality

**FIGURE 6:**
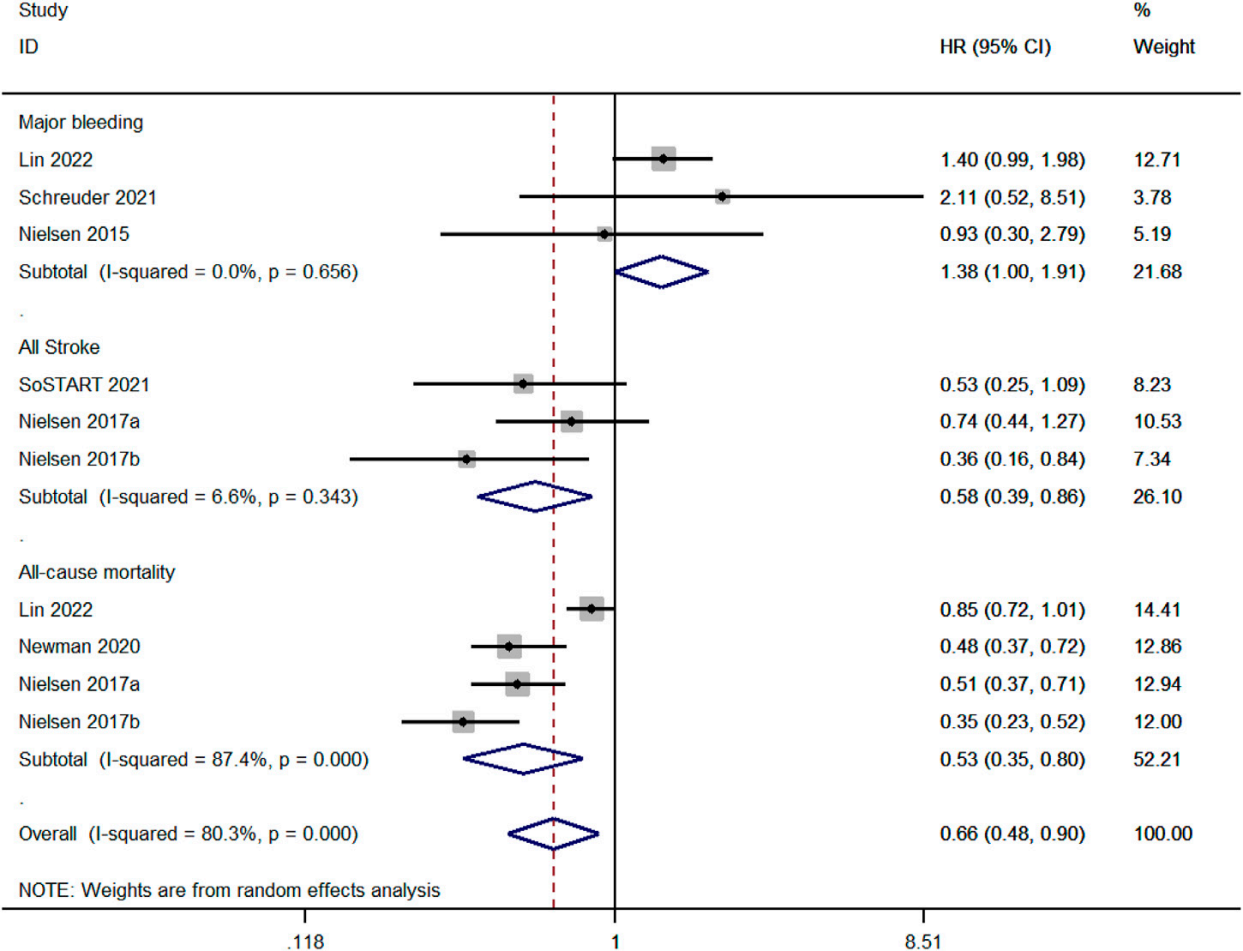
Meta-analysis of the risk of major bleeding in OAC *versus* non-OAC.

We compared the risk of major bleeding between OAC and non-OAC in three studies (Nielsen et al., 2015; Schreuder et al., 2021; Lin et al., 2022). There was no heterogeneity among the studies (*p* ≥ 0.1 and I^2^ ≤ 50%), and the fixed-effect model was performed. The risk of major bleeding for OAC was higher than non-OAC (aHR, 1.38; 95%, 1.00–1.91, *p* = 0.03). Three studies compared the risk of all strokes between OAC and non-OAC (Nielsen et al., 2017; SoSTART Collaboration, 2021). There was no heterogeneity among the studies (*p* ≥ 0.1 and I^2^ ≤ 50%), and the fixed-effect model was performed. The risk of all strokes for OAC therapy was lower than non-OAC (aHR, 0.67; 95%, 0.59–0.77, *p* < 0.001). Four studies compared the all-cause mortality between OAC and non-OAC (Nielsen et al., 2017; Newman et al., 2020; Lin et al., 2022). There was high heterogeneity among the studies (*p* < 0.1 and I^2^> 50%), and the random-effect model was applied. OAC had a lower risk of all-cause mortality than non-OAC (aHR, 1.38; 95%, 1.00–1.91, *p* = 0.03, Figure 6).

### Comparing the effectiveness and safety of DOACs *versus* warfarin

Three studies evaluated the effectiveness and safety between DOACs and warfarin (Lee et al., 2020; Tsai et al., 2020; Lin et al., 2022). Major bleeding risk was similar between the two groups (aHR, 0.54; 95%, 0.26–1.10, *p* = 0.088). However, DOACs significantly reduced risks for Ischemic Stroke/SE (aHR, 0.84; 95%, 0.70–1.00, *p* = 0.049), recurrent ICH (aHR, 0.63; 95%, 0.49–0.82, *p* = 0.001) and all-cause mortality (aHR, 0.65; 95%, 0.48–0.88, *p* = 0.005, Table 3) compared to warfarin.

**TABLE 3:** Results of Comparison effectiveness and safety of DOACs *versus* warfarin.

Outcomes	Number of studies/patients	I^2^ (%)	Meta-analysis results	
			aHR (95% CI)	*p*-value
Ischemic Stroke/SE	3/8,188	0	0.84 (0.70–1.00)	0.049
Major bleeding	2/2,484	84.2	0.54 (0.26–1.10)	0.088
Recurrent ICH	3/8,188	0	0.63 (0.49–0.82)	0.001
All-cause mortality	3/8,188	82	0.65 (0.48–0.88)	0.005

### Subgroup analyses based on region and type of study for OAC *versus* non-OAC

Only the primary outcomes of OAC *versus* non-OAC were compared in subgroups due to the limited number of included studies comparing DOACs with warfarin (three articles). In non-Asian regions, OAC and non-OAC therapy had similar results for recurrent ICH (aHR, 0.93; 95% CI, 0.51–1.69, *p* = 0.81) and cohort studies (aHR, 0.96; 95% CI, 0.57–1.59, *p* = 0.861). However, OAC indicated an increased risk of recurrent ICH in the Asian region (aHR, 1.51; 95% CI, 1.23–1.86, *p* < 0.001) compared to non-OAC. OAC indicated an increased risk in RCTs, but the difference was not statistically significant (aHR, 2.73; 95% CI, 0.95–7.89, *p* = 0.063) compared to non-OAC. For Ischemic Stroke/SE, OAC significantly reduced risks in Asia (aHR, 0.59; 95% CI, 0.48–0.72, *p* < 0.001), Non-Asian (aHR, 0.74; 95% CI, 0.58–0.94, *p* = 0.016) and cohort studies (aHR, 0.64; 95% CI, 0.55–0.74, *p* < 0.001) compared to non-OAC. Supplementary Table S1 describes the subgroup analysis of the included studies.

### Sensitivity analysis and publication bias

In the sensitivity analysis, all studies would be excluded sequentially. Primary outcome indicators remained unchanged, indicating that our meta-analysis yielded stable results (Supplementary Table S2). Funnel plots were not employed to assess publication bias due to the small number of included studies (less than 10).

## Discussion

ICH, one of the leading causes of disability and death worldwide, is caused by a rupture of blood vessels in the brain parenchyma or subarachnoid space. ICH is the most dangerous complication of OAC therapy in AF patients; the annual rate is 0.6%–1.0% (Qureshi et al., 2009; Steiner et al., 2014). Additionally, OAC therapy-associated ICH tends to bleed more severely and is associated with higher mortality than spontaneous ICH (Li and Lip, 2018). The latest guidelines do not provide strong recommendations on which anticoagulation regimen (non-OAC or warfarin or DOACs) to use for patients with AF who have ICH (Greenberg et al., 2022).

Previous studies have consistently demonstrated that antiplatelet therapy after ICH does not reduce the risk of ischemic stroke and increases the risk of recurrent ICH (Nielsen et al., 2015; Lin et al., 2022). The antiplatelet treatments have no clinical benefit in the treatment of AF who have a history of ICH, and such studies were eliminated from all further analyses. Whether patients with AF who have ICH require anticoagulation is controversial. OAC therapy was associated with a lower risk of thromboembolic events and a similar risk of ICH recurrence in an earlier meta-analysis based on retrospective studies (Murthy et al., 2017). Although this study was a high-quality meta-analysis, it had two important limitations. The study included patients with AF, mechanical heart valve, and venous thrombosis, which may inevitably introduce bias in some outcomes. Additionally, there were no RCTs in the included studies, and most of the observational studies had some design flaws. We collected seven cohort studies and two RCTs to evaluate the benefits and risks of OAC treatment using aHRs.

The study results revealed that OAC therapy significantly reduced the risk of Ischemic Stroke/SE and all-cause death after ICH compared to non-OAC therapy but did not increase the risk of recurrent ICH. The results suggest a greater clinical benefit of starting anticoagulation after ICH. Although four studies compared the risk of all-cause death between the two groups, brain imaging or autopsies were unavailable in observational studies, so it was unclear whether the death was due to major bleeding, ischemic stroke, or something else. Therefore, the conclusions should be interpreted with caution. DOACs have become the first choice in non-valvular AF but remain uncertain in AF patients with a history of ICH (Wadhera et al., 2014). Our research found that DOACs were associated with a lower risk of Ischemic Stroke/SE, recurrent ICH, and all-cause death. The conclusion is consistent with the study of Suah BH et al. They revealed that DOACs were superior to warfarin in efficacy and safety when the anticoagulation was started (Suah et al., 2022). Asian patients with AF on OAC therapy have a higher risk of ICH than patients in other regions. This may cause clinicians to hesitate to restart or initiate anticoagulation (Chiang et al., 2014). In the subgroup analysis, we discovered that anticoagulation therapy did not increase the risk of recurrent ICH in non-Asian populations but increased the risk of recurrent ICH in Asian populations. The results indicated that the starting of OAC therapy did not necessarily beneficial for all populations. The results also vary by study type, with higher rates of recurrent ICH in RCTs involving OAC than those involving non-OAC but no difference in recurrent ICH rates between groups in cohort studies. The possible reason is that the patients who were started on anticoagulation therapy had a low risk of ICH recurrence or smaller hematomas in the retrospective studies after clinical assessment. However, there were strict inclusion and exclusion criteria in the RCTs, without the subjective judgment of the clinician. In comparison, the RCTs had a higher level of evidence and their findings had a higher clinical value. Our result suggests that initiation of anticoagulation after ICH may increase the risk of recurrent ICH, and the decision needs to be made by the clinician. If the patient is found to have a lower risk of recurrent ICH, then anticoagulation therapy will be initiated. If the patient is found to have a higher risk of ICH, left atrial appendage (LAA) occlusion will be proposed as an alternative to anticoagulation (Holmes et al., 2014).

We are aware that this systematic review has some limitations. This study included only two RCTs, all were small sample studies, and the rest were cohort studies. Therefore, this conclusion must be verified by additional RCTs. The optimal timing for starting OAC after ICH cannot be determined because the timing varies between studies. We did not evaluate the superiority or inferiority of the various DOACs due to the small sample size. If ICH was classified according to the cause of disease, lobar ICH was more likely secondary to amyloid angiopathy, and the ICH event recurrence rates were twice that of non-lobar ICH recurrence (Kuramatsu and Huttner, 2019). As a result, the prognosis varies depending on the type of ICH. The diagnosis of ICH was based on a diagnostic code in the retrospective studies. The location and type of ICH were not determined due to an inability to image the brain; therefore, we did not perform a subgroup analysis of the different types of ICH. The results may be overestimated or underestimated due to the lack of information regarding the intensity of warfarin treatment in the observational studies.

## Conclusion

In summary, the risk of Ischemic Stroke/SE was lower with OAC than non-OAC in patients with a history of AF and ICH and did not increase the risk of recurrent ICH. Additionally, DOACs were superior to warfarin regarding efficacy and safety when choosing between anticoagulants. In Asia, the risk of recurrent ICH was higher with OAC than with non-OAC. This meta-analysis suggested that this clinical decision-making applies to OAC in AF and ICH patients in non-Asian populations.
