# Supplementary material for: Efficacy and safety of anticoagulation in atrial fibrillation patients with intracranial hemorrhage: A systematic review and meta-analysis
Source: Front Pharmacol. 2023 Mar 9;14:1122564. doi: 10.3389/fphar.2023.1122564 (PMC10033967; doi:10.3389/fphar.2023.1122564)
Supplement: Supplementary file 1 [file Table1.docx]

SUPPLEMENTAL FILE

Title: Efficacy and safety of anticoagulation in atrial fibrillation patients with intracranial hemorrhage: A systematic review and meta-analysis

**Supplementary Table 1. Results of Subgroup Analysis**

| **Recurrent ICH** | **No. of studies/**  **patients** | **Meta-analysis results** | | **Ischemic Stroke/SE** | **No. of studies/**  **patients** | **Meta-analysis results** | |
| --- | --- | --- | --- | --- | --- | --- | --- |
|  |  | **aHR（95% CI）** | ***P*-value** |  |  | **aHR（95% CI）** | ***P*-value** |
| **Region** |  |  |  |  |  |  |  |
| Asia | 2/3,620 | 1.51 (1.23-1.86) | < 0.001 | Asia | 2/3,620 | 0.59 (0.48-0.72) | < 0.001 |
| Non-Asian | 4/4,221 | 0.93 (0.51-1.69) | 0.81 | Non-Asian | 4/4,221 | 0.74 (0.58-0.94) | 0.016 |
| **Type of study** |  |  |  |  |  |  |  |
| RCT | 2/304 | 2.73 (0.95-7.89) | 0.063 | RCT | 1/101 | NS | NS |
| Cohort studies | 4/7,537 | 0.96 (0.57-1.59) | 0.861 | Cohort studies | 4/7,537 | 0.64 (0.55-0.74) | < 0.001 |

**Supplementary Table 2. Sensitivity analysis of studies**

| **Omitted Recurrent ICH** | **aHR (95% CI)** | **Omitted Ischemic Stroke/SE** | **aHR (95% CI)** |
| --- | --- | --- | --- |
| Lin SY 2022 | 1.08 (0.60–1.92) | Lin SY 2022 | 0.65 (0.55–0.77) |
| Schreuder 2021 | 1.02 (0.62–1.67) | Schreuder 2021 | 0.64 (0.55–0.74) |
| SoSTART 2021 | 0.99 (0.60–1.65) | Newman 2020 | 0.58 (0.48–0.70) |
| Newman 2020 | 1.21 (0.72–2.01) | Nielsen 2017a | 0.65 (0.56–0.76) |
| Nielsen 2017a | 1.05 (0.60–1.84) | Nielsen 2017b | 0.65 (0.55–0.76) |
| Nielsen 2017b | 1.25 (0.80–1.93) | Chao 2016 | 0.70 (0.57–0.86) |
| Chao 2016 | 0.95 (0.58–1.56) |  |  |
